# 

**Published:** 1895-03

**Authors:** 


### American Medical Publishers' Association.

The annual meeting of the American Medical Publishers' Association will be held in Baltimore, on May 6th, convening in the parlors of the Eutaw House, at 9:30 a. m. An interesting programme is being prepared, to which publishers are invited to contribute in the way of a paper on some subject connected with the medical publishing business.

Medical journalists should make the Baltimore meeting a representative one, and thus demonstrate the value of the Association.

